# A Brain-Computer Interface Based on Bilateral Transcranial Doppler Ultrasound

**DOI:** 10.1371/journal.pone.0024170

**Published:** 2011-09-07

**Authors:** Andrew J. B. Myrden, Azadeh Kushki, Ervin Sejdić, Anne-Marie Guerguerian, Tom Chau

**Affiliations:** 1 Bloorview Research Institute, Holland Bloorview Kids Rehabilitation Hospital, Toronto, Ontario, Canada; 2 Institute of Biomaterials and Biomedical Engineering, University of Toronto, Toronto, Ontario, Canada; 3 Department of Electrical and Computer Engineering, University of Pittsburgh, Pittsburgh, Pennsylvania, United States of America; 4 Departments of Critical Care Medicine and Pediatrics, The Hospital for Sick Children, Toronto, Ontario, Canada; 5 Neuroscience and Mental Health Research Program, Hospital for Sick Children Research Institute, Toronto, Ontario, Canada; 6 Institute of Medical Science, University of Toronto, Toronto, Ontario, Canada; Georgia State University, United States of America

## Abstract

In this study, we investigate the feasibility of a BCI based on transcranial Doppler ultrasound (TCD), a medical imaging technique used to monitor cerebral blood flow velocity. We classified the cerebral blood flow velocity changes associated with two mental tasks - a word generation task, and a mental rotation task. Cerebral blood flow velocity was measured simultaneously within the left and right middle cerebral arteries while nine able-bodied adults alternated between mental activity (i.e. word generation or mental rotation) and relaxation. Using linear discriminant analysis and a set of time-domain features, word generation and mental rotation were classified with respective average accuracies of 82.9%
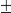
10.5 and 85.7%
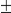
10.0 across all participants. Accuracies for all participants significantly exceeded chance. These results indicate that TCD is a promising measurement modality for BCI research.

## Introduction

Brain-computer interfaces (BCIs) translate mental activity into control signals for external devices, thereby providing their users with movement-free communication and control channels [1]. BCIs can be employed in a wide variety of areas including virtual reality [2], neurorobotics [3], and wheelchair control [4]. There has also been a great deal of research into the usage of BCIs as means of communication and control for individuals with severe and multiple disabilities [5], [6]. BCI systems offer these individuals the potential to achieve some degree of independence and control over their environments. Moreover, BCI control bypasses the muscular system entirely and thus may allow communication even for those who are completely locked-in due to conditions such as stroke or amyotrophic lateral sclerosis (ALS) [7]. For instance, BCIs have been used to generate text communication based on measuring brain responses to visually presented letters on a computer screen [8].

In BCI systems, mental activity can be detected using various measurement modalities including electroencephalography (EEG) [9], functional magnetic resonance imaging (fMRI) [10], magnetoencephalography (MEG) [11], and near-infrared spectroscopy (NIRS) [12]. Though BCI systems developed using these modalities have shown promise in controlled environments, their practical success has been limited by a number of shortcomings [13]. The most commonly used measurement modality in BCI systems is EEG. These signals are susceptible to interference from electrical sources and physiological artifacts such as electrooculography (EOG) and electromyography (EMG) [5]. Moreover, proficient use of EEG-based BCIs often requires several training sessions. BCI systems based on fMRI and MEG measurements employ extremely expensive instruments and require highly controlled environments [14]. Consequently, these technologies are presently impractical for widespread use [15]. NIRS is still early in its development as a BCI technology. Current studies employing this modality have predominantly focused on the slow hemodynamic response, resulting in low data transmission rates [16]. These shortcomings of current BCI systems motivate the investigation of alternative measurement modalities.

In light of the above limitations, this paper investigates transcranial Doppler (TCD) sonography as the foundation for a new type of non-invasive BCI. TCD is a medical imaging technique used to monitor cerebral blood flow velocity (CBFV) within the major arteries of the circle of Willis - namely the anterior, middle, and posterior cerebral arteries [17]. Since its introduction in 1982 [18], TCD has been successfully used in a number of clinical applications [19]–[23]. It has also been used extensively to describe brain function through the study of cerebral lateralization [24]–[28]. TCD is portable, lightweight, and robust to environmental conditions such as electrical artifacts [29]. It is also relatively inexpensive, particularly in comparison to alternatives such as fMRI and MEG [30]. TCD possesses excellent temporal resolution, and previous research into lateralization indicates that event-related changes in CBFV can be observed within 5–10 seconds of the onset of cognitive activity in some cases [30], [31]. Most importantly, cognitive activation produces increases in CBFV [32] that are easily measurable by TCD.

The above features suggest that TCD may be a suitable measurement modality for BCI systems. To further demonstrate the viability of a TCD-based BCI system, it must be shown that mental activity can be automatically detected with high accuracy based on these measurements. In this light, the present study investigated whether two specific mental tasks can automatically be differentiated from rest using TCD measurements. If adequate classification accuracies can be obtained, these mental tasks can be used to generate control signals in a TCD-based BCI system.

We investigated two mental tasks, namely word generation and mental rotation of geometric shapes. Word generation is known to cause significant increases in CBFV within the left and right middle cerebral arteries (MCAs) [24], [25]. Moreover, these increases have been characterized as being left-lateralized in right-handed individuals - higher relative increases in CBFV have been found in the left MCA than in the right MCA [33]. We expected that this lateralization would make it possible to automatically detect the word generation activity. Spatial tasks involving mental rotation have also been explored in a number of TCD studies [25], [34,25], where they have likewise been observed to cause significant increases in CBFV within the left and right MCAs. We hypothesized that large bilateral increases in CBFV induced by complex mental rotation tasks can be automatically detected using TCD measurements.

## Materials and Methods

### Ethics Heading

This study was approved by the Research Ethics Boards of both Holland Bloorview Kids Rehabilitation Hospital and the University of Toronto. All participants provided written informed consent.

### Participant

Nine able-bodied participants (6 female) were recruited from amongst the population of the Bloorview Research Institute. Ages of the participants at the time of the study ranged between 22 and 30 (mean age 25.6 years). All participants were right-handed, as quantified by the Edinburgh Handedness Inventory [36], with scores ranging between 50 and 100 (mean score 79.4). Participants had no history of migraine, and no known neurological or respiratory conditions.

### Signal Acquisition

CBFV was monitored using a Multi-Dop X4 TCD unit (Compumedics USA). Dual 2 MHz ultrasonic transducers were fitted on the included headgear and placed over the left and right transtemporal windows, located in front of the ear and above the zygomatic arch [33]. The headgear ensured that the transducers remained stationary throughout the experiment. For all participants, a screening test was performed to ensure that there were no CBFV abnormalities. Following the insonation procedure detailed by Alexandrov et al. [37], CBFV was measured within the left and right anterior, middle, and posterior cerebral arteries. Compared to expected velocities [38], no unusual values were observed, and thus all participants were accepted for this study. Optimal transducer locations for each participant were determined during the screening test and recorded by the experimenter. During subsequent sessions, transducers were placed at the previously recorded locations to ensure within-participant consistency and repeatability. The same procedure was used for all participants. Locations were similar but not identical between participants, due to slight deviations in the location of the transtemporal window.

Each participant completed two sessions. Insonation followed the same procedure that was used for the screening test. We measured CBFV within the left and right middle cerebral arteries (MCAs). These arteries were selected because the MCAs profuse approximately 80% of the brain [28] and, as such, are implicated in a wide variety of mental tasks [25]. Signals were acquired by adjusting the probe angle, probe location, and measurement depth until optimal signals were located. Insonation depths ranged from 45 to 60 mm. Signals were acquired from approximately the same depth on both sides. Signals from both channels - the left MCA and the right MCA - were used to characterize each state during the experiment. Thermal cranial index was monitored and kept below 2 at all times. The lowest power level for which signals were adequate was used. Insonation lasted for no longer than 15 minutes at a time, consistent with ultrasound safety guidelines such as [39].

Respiratory modulation and carbon dioxide levels are known to influence CBFV [40]. These signals were recorded to ensure that fluctuations in CBFV did not simply result from changes in respiration. Participants wore a nasal cannula, which was connected to a capnometer built into the Multi-Dop X4 unit to monitor end-tidal CO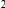 levels. Respiration was also directly measured using a respiratory belt. Blood volume pulse (BVP) was measured using an FDA-approved photoplethysmography sensor (Flexcomp Infiniti, Thought Technologies Ltd.). The sensor was secured to the palmar surface of the distal phalange of the first digit of the non-dominant hand.

### Experimental Protocol

At the beginning of each session, participants were seated comfortably facing a computer monitor in a data collection room. Following signal acquisition, participants rested naturally for 10 minutes to allow CBFV to stabilize. Data from this baseline period were not used for further analysis. Following this interval, participants received verbal instructions on how to perform the two required mental tasks. Participants were then instructed to begin the experiment when ready.

In each session, participants completed two 15-minute blocks, separated by a 5-minute break. Each block consisted of 10 rest periods and 10 activation periods. Each period had a duration of 45 seconds, and successive periods alternated between rest and activation states. During activation states, participants received onscreen prompts to perform either the word generation or mental rotation task. Each task occurred five times within each block in randomized order. Each block proceeded automatically once begun, and included only text-based prompts. There were no auditory distractions during the experiment. The experiment contained a total of 40 rest states, 20 word generation states, and 20 mental rotation states for each participant.

During the word generation task, a letter was presented on screen and participants were prompted to silently generate words that began with the given letter. Letters were selected from among the most common first letters of English words. No letters were repeated within sessions, but some letters were used in both sessions.

During the mental rotation task, participants were presented with four pairs of figures simultaneously. Each pair consisted of two similar objects rotated to different angles around the x-axis. Participants were required to mentally rotate the two objects in each pair to determine if they were the same object or mirror images. Participants were instructed to work sequentially through all four pairs. Each pair was randomly selected from a database of such figures [41]–[43]. The entire set of figures was replaced with four new pairs every nine seconds. Post-experiment feedback from participants confirmed that this method allowed them to constantly perform this task over the entire activation period.

Participants were instructed to keep their eyes open during both activation and rest, and to perform each task as quickly as possible. Participants were also instructed to refrain from vocalizing their answers, thus preventing CBFV increases due to speech. During rest periods, participants were instructed to relax. No instructions were given regarding modulation of respiration.

### Pre-processing

TCD data were exported from the Multi-Dop X4, and the mean of the maximum velocity was extracted for analysis. This parameter is automatically computed by the Multi-Dop X4, and reduces the effect of CBFV variability between systole and diastole. The raw data from each block were normalized, and then filtered using a third-order Butterworth low-pass filter with a cutoff frequency of 0.6 Hz to remove the effects of beat-to-beat fluctuations in CBFV. The data were then segmented into rest and activation states, using markers that were automatically inserted into the TCD recordings at the beginning of each state during the experiment. Data from the respiratory belt were also segmented into rest and activation states.

### Feature Extraction

Feature extraction was performed on the recorded signals from each rest and activation state. The list of extracted features is given in Table 1. All features were computed over four intervals within each state - 0–45 seconds, 0–15 seconds, 15–30 seconds, and 30–45 seconds. Features were divided between unilateral features, which were dependent solely on the signal from one MCA, and bilateral features, which compared signals from both MCAs. For respiratory signals, we extracted the signal mean and the respiration rate.

**Table 1:** Candidate feature set.

Feature Number	Feature Description	Laterality
1–4	Left MCA Mean (LM)	Unilateral
5–8	Left MCA Slope (LS)	Unilateral
9–12	Left MCA Standard Deviation (LSD)	Unilateral
13–16	Right MCA Mean (RM)	Unilateral
17–20	Right MCA Slope (RS)	Unilateral
21–24	Right MCA Standard Deviation (RSD)	Unilateral
25–28	Lateralization (Difference of Means) (DM)	Bilateral
29–32	Lateralization (Difference of Slopes) (DS)	Bilateral
33–36	Cross-correlation of Left and Right MCAs (CC)	Bilateral
37–40	Dot Product of Left and Right MCAs (DP)	Bilateral

All features were computed over four different time intervals - 0–45 s, 0–15 s, 15–30 s, and 30–45 s.

### Feature Selection

Features were selected on the basis of the Fisher criterion [44]. For one feature, this criterion can be expressed as:
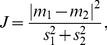
(1)where 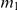 and 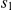 represent the mean and standard deviation of a feature evaluated over all rest states, and 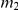 and 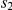 the mean and standard deviation of the same feature evaluated over all activation states. The Fisher criterion increases as the average separation between groups increases and the average separation within groups decreases. Using this criterion for feature selection yields the features that provide maximum separability between rest and activation patterns.

Using only the Fisher criterion for feature selection could potentially lead to the selection of highly correlated features. When multiple features were required, the feature set was first reduced by selecting the eight highest-ranking features based on the Fisher criterion. The highest-ranked feature was selected as the initial feature. To select subsequent features, the correlation coefficients between the initially selected feature and each remaining feature were computed. The feature with the lowest magnitude correlation coefficient was then selected. If necessary, a similar procedure was used to select a third feature, taking into account the correlation with both previously selected features.

### Classification

Twenty runs of five-fold cross-validation were performed for n = 1, 2, and 3 features. A linear discriminant analysis (LDA) classifier was used. Both activation states (i.e. mental rotation and word generation) were compared independently to the rest state using the same procedure. Due to unbalanced classes, 20 rest states were randomly selected at the beginning of each run to be used during classification. During each fold, feature selection was performed using only training data.

Classification was also performed using several reduced sets of features. Namely, classification was performed using only features from the respiratory belt, and only bilateral features from the TCD recordings. In the latter case, three features were selected from the set of bilateral features using the procedure already described.

### Evaluation Criteria

The percentage of correctly classified samples was used as the evaluation criteria. We also report sensitivity (the percentage of correctly classified activation states) and specificity (the percentage of correctly classified rest states).

## Results

**Figure 1:**
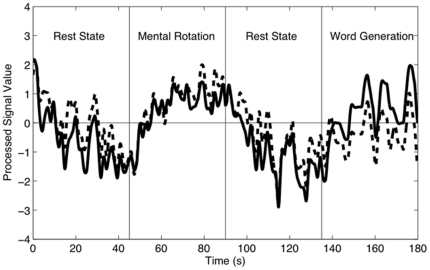
Recordings from two rest-activation cycles for participant 4. The solid line depicts CBFV in the left MCA, while the broken line depicts CBFV in the right MCA. Decreasing trends in CBFV during rest and increasing trends during activation are apparent. The signal is the mean of the maximum velocity, filtered by a Butterworth low-pass filter with a cutoff frequency of 0.6 Hz.

A sample TCD recording depicting CBFV fluctuations during a three-minute segment of the experiment is shown in Figure 1. In this recording, visually detectable differences between rest and activation states are apparent. In particular, we note apparent bilateral activation during the mental rotation task, and left lateralization during the word generation task.

**Figure 2:**
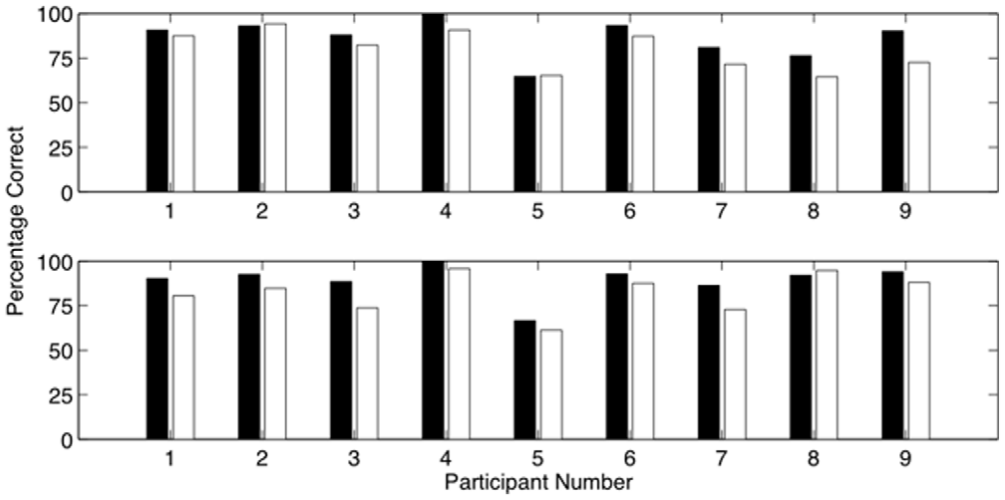
Sensitivities and specificities for all participants for both tasks. The word generation task is on top, and the mental rotation task on bottom. Black bars correspond to sensitivity, and white bars to specificity. Sensitivity was significantly greater than specificity (Wilcoxon rank-sum test, p
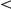
0.03) for six of nine participants for word generation (exceptions are Participants 1, 2, and 5) and for seven of nine participants for mental rotation (exceptions are Participants 5 and 8).

The accuracies for each task, participant, and feature selection condition are reported in Tables 2 and 3. The best results were achieved using three features (mean classification accuracy for word generation and mental rotation tasks were 82.9
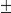
10.5% and 85.7
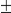
10.0% respectively). For this case, sensitivities and specificities are reported in Figure 2.

**Table 2:** Classification accuracies for the word generation task.

Participant Number	CBFV Only (1 Feature)	CBFV Only (2 Features)	CBFV Only (3 Features)	Bilateral Features Only	Respiration (2 Features)
1	87.9 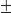 11.2	89.5 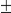 10.2	89.1 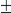 11.6	87.5 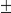 11.2	69.6 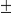 16.4
2	94.4 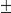 7.0	91.9 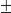 9.5	93.6 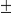 8.6	94.6 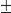 7.8	50.8 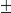 16.3
3	82.6 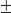 12.3	82.9 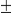 12.4	85.1 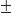 12.3	87.3 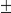 10.8	52.8 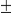 15.8
4	88.5 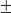 9.7	94.2 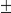 8.0	95.3 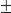 6.6	89.8 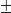 10.0	85.9 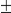 11.6
5	61.5 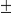 15.2	64.1 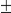 17.4	65.0 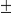 18.6	64.5 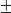 16.7	55.9 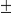 17.4
6	87.1 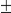 11.4	87.5 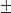 12.9	90.2 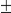 10.6	88.4 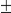 9.4	71.1 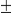 14.0
7	71.3 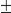 14.9	73.3 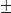 15.7	76.3 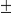 14.3	70.3 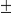 15.2	70.8 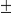 14.5
8	68.5 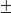 15.1	70.5 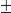 15.4	70.4 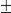 15.7	70.5 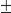 14.9	71.0 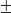 15.6
9	74.0 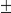 16.1	79.2 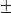 12.2	81.4 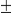 12.5	83.8 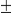 11.7	70.3 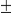 14.2
Average	79.5 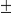 11.1	81.5 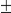 10.4	82.9 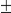 10.5	81.8 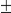 10.6	66.4 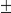 11.2

Columns two through four show accuracies when 1–3 features were selected from the entire candidate pool. Column 5 shows the accuracies when the candidate pool was restricted to the 16 bilateral features. In this case, the analysis was performed for only the selection of three features, using the same feature selection algorithm. The final column shows classification accuracies when only respiratory features were used. All participants except for Participant 8 displayed significantly higher accuracies for three-feature TCD classification (using the entire candidate pool) than for classification based on respiration. Mean classification accuracy was significantly greater for two and three features than for one feature (repeated-measures regression, p
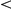
0.012). The comparison between two and three features approached significance (p = 0.056).

**Table 3:** Classification accuracies for the mental rotation task.

Participant Number	CBFV Only (1 Feature)	CBFV Only (2 Features)	CBFV Only (3 Features)	Bilateral Features Only	Respiration (2 Features)
1	79.8 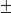 12.5	83.1 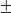 11.7	85.4 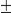 10.7	74.9 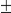 13.0	74.5 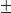 14.0
2	82.0 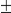 12.1	86.9 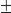 11.4	88.6 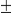 11.1	74.4 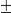 16.0	79.5 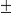 13.0
3	77.1 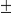 15.4	80.3 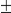 12.8	81.1 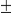 13.0	70.8 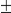 13.3	63.8 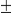 16.9
4	95.0 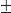 7.5	96.1 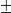 6.6	97.9 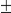 4.7	57.3 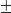 15.9	82.0 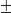 12.7
5	56.5 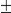 16.7	59.0 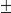 14.8	63.9 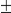 15.2	58.9 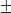 14.3	55.1 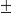 18.9
6	90.3 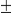 10.9	89.8 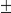 10.0	90.1 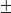 10.3	64.4 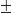 17.4	88.0 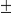 11.5
7	78.3 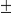 12.5	81.3 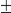 14.1	79.5 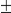 12.8	56.1 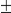 16.4	65.5 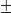 16.8
8	91.9 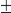 9.6	92.8 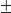 8.7	93.4 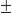 9.0	92.9 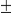 8.6	70.3 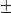 14.2
9	85.6 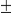 11.7	91.5 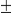 8.5	91.0 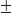 9.1	54.9 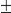 18.3	82.2 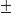 12.7
Average	81.8 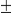 11.4	84.5 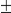 11.0	85.7 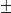 10.0	67.2 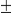 12.4	73.4 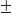 10.6

Columns two through four show accuracies when 1–3 features were selected from the entire candidate pool. Column 5 shows the accuracies when the candidate pool was restricted to the 16 bilateral features. In this case, the analysis was performed for only the selection of three features, using the same feature selection algorithm. The final column shows classification accuracies when only respiratory features were used. All participants except for Participant 6 displayed significantly higher accuracies for three-feature TCD classification (using the entire candidate pool) than for classification based on respiration. Mean classification accuracy was significantly greater for two and three features than for one feature (repeated-measures regression, p
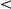
0.001). There was no significant difference between two and three features.

Chance results were simulated by performing classification with randomized state labels, resulting in accuracies of approximately 50%. Results from three-feature classification were compared to these chance results using the Wilcoxon rank sum test at a 0.05 significance level. These results were also compared to those from classification of respiratory signals using the same procedure. All participants showed accuracies that were significantly greater than chance (p
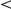
0.0001) for both tasks. Eight of nine participants showed a significant difference between CBFV and respiration classification for both tasks (p
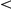
0.015).

**Figure 3:**
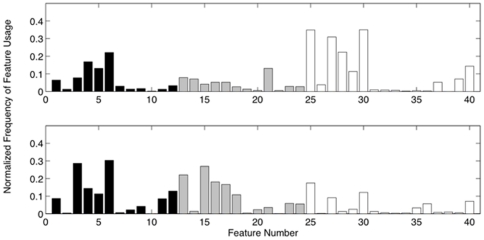
Average feature selection across all participants for both tasks. The word generation task is on top, and the mental rotation task on bottom. Black bars are left MCA features, grey bars are right MCA features, and white bars are bilateral features. Bilateral features are more frequently selected for the word generation task, likely due to the left-hemispheric lateralization of this task. Feature descriptions can be found in Table 1.

**Figure 4:**
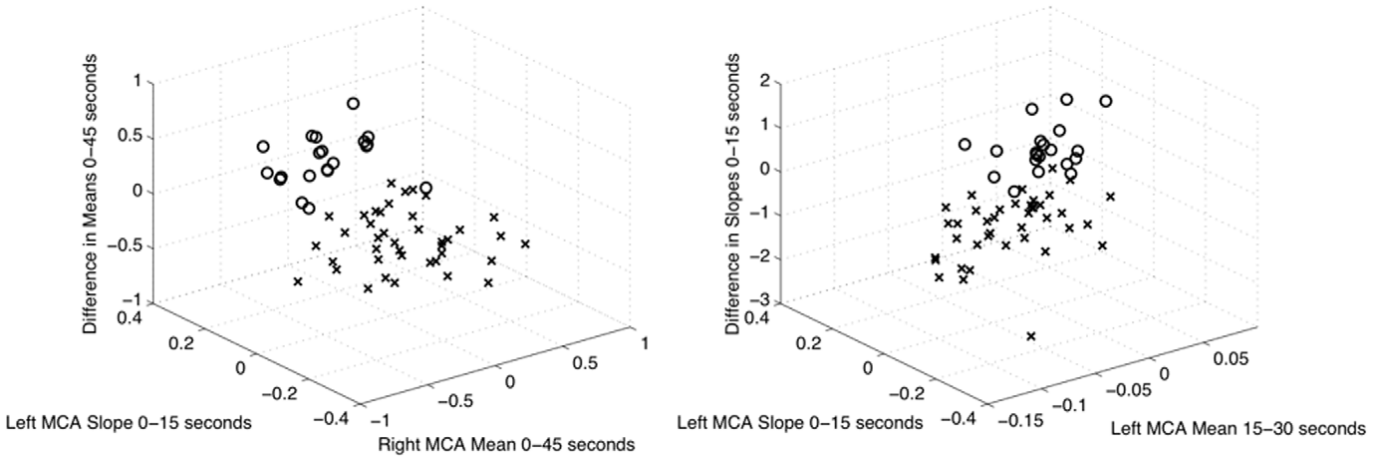
Scatter plots showing the relationship between rest and activation for Participant 2. The word generation task is on the left, and the mental rotation task on the right. The three most frequently selected features for each task have been used to characterize each state. Rest and activation states are represented by ‘x’ and ‘o’ respectively. Both tasks are highly separable for this participant.

The most frequently selected features for each participant are given in Table 4, while average feature selection across all participants is shown in Figure 3. A sample scatter plot based on the three most frequently selected features for Participant 2 is shown in Figure 4.

**Table 4:** Most frequently selected features for each participant for each task (abbreviations given in Table 1).

Participant Number	Rank	Word Generation	Mental Rotation
	1	DM 30–45 sec	DM 0–45 sec
1	2	DS 0–15 sec	RM 0–45 sec
	3	RM 0–15 sec	RM 15–30 sec
	1	LS 0–15 sec	LM 15–30 sec
2	2	DM 0–45 sec	LS 0–15 sec
	3	RM 0–45 sec	DS 0–15 sec
	1	DM 0–45 sec	RM 30–45 sec
3	2	RSTD 0–45 sec	LS 0–15 sec
	3	LS 0–15 sec	RM 0–45 sec
	1	LM 30–45 sec	LM 30–45 sec
4	2	LS 0–15 sec	LS 0–15 sec
	3	DM 30–45 sec	RM 15–30 sec
	1	LM 0–45 sec	DP 30–45 sec
5	2	DM 30–45 sec	CC 30–45 sec
	3	DS 0–15 sec	CC 15–30 sec
	1	DM 15–30 sec	RM 15–30 sec
6	2	DP 15–30 sec	LS 0–15 sec
	3	DS 0–15 sec	LM 15–30 sec
	1	DS 0–45 sec	RM 0–45 sec
7	2	LS 0–45 sec	LM 15–30 sec
	3	DM 15–30 sec	LS 0–45 sec
	1	DP 30–45 sec	DS 0–15 sec
8	2	DM 0–45 sec	DM 0–45 sec
	3	DM 15–30 sec	DM 15–30 sec
	1	DM 0–45 sec	DS 0–45 sec
9	2	DS 0–15 sec	DS 0–15 sec
	3	RSTD 0–45 sec	DM 0–45 sec

## Discussion

### Feasibility of a TCD-based BCI

This study investigated the potential of TCD as the measurement modality for a BCI. We demonstrated that two types of mental activity can be classified with greater than 80% accuracy on the basis of changes in cerebral blood flow velocity. These accuracies were achieved without any prior training. It is likely that this can be partially attributed to the usage of intuitive mental tasks as activation states. Our results show that TCD is a promising measurement modality for BCI development, and further research should be performed to continue investigation into the performance of a TCD BCI.

### Classification of Word Generation and Mental Rotation

For both mental tasks, we observed that sensitivity was generally higher than specificity (see Figure 2). This may be related to the more specific directions given for activation states compared to rest states. During activation, participants performed one of two well-defined tasks. During rest, participants were simply instructed to relax - they were not instructed to ‘think of nothing’ or to perform any specific low-intensity mental task. Therefore, it seems reasonable for rest states to be less consistent than activation states, leading to lower specificities. This usage of relaxation as a rest state reflects realistic conditions for BCI use.

Some participants displayed large variations in accuracy between the two tasks. Most glaringly, Participant 8 attained 70% accuracy for the word generation task, but 93% accuracy for the mental rotation task. Such individual differences highlight the importance of appropriate task selection. In a TCD-based BCI that is operated using simple mental tasks, significant gains in performance could potentially be achieved by testing a number of different mental tasks and choosing the optimal task on a case-by-case basis.

One individual, Participant 5, had accuracies which were considerably lower than average. For this individual, the transtemporal insonation window was very difficult to find. Although adequate TCD signals were acquired and recorded during each session, it is possible that the extended set-up time and associated frustration for the participant were partially responsible for these lower accuracies.

The mental rotation task was classified with a slightly higher accuracy than the word generation task. However, the word generation task is considerably easier to comprehend and perform, and may be more suitable for a younger population. The word generation task also has a significant advantage in that, with practice, it can be performed autonomously without any prompting. This ease of implementation means that the word generation task may be a more promising alternative for future TCD-based BCI development.

### Feature Selection

During classification, we used two main types of features - unilateral features and bilateral features. Unilateral features are important when a signal parameter shows significant differences between activation and rest. Selection of these features may reflect a difference in net cognitive load between rest and activation states. Bilateral features are important when a given mental task causes some level of asymmetry between activation in the left and right hemispheres of the brain. As such, selection of these features may represent a difference in lateralization between rest and activation states. From this reasoning, it seems plausible that markedly different features could be selected for the classification of different mental tasks. Indeed, this proved to be the case in this study, as seen in Table 4. However, for each task, there was some consistency across all participants.

Consistent with previous findings [25], we observed that the word generation task was strongly lateralized to the left MCA in most participants. Consequently, bilateral features were frequently selected. More specifically, the features corresponding to differences in means and differences in slopes were very important during this task; two of the three most commonly selected features came from these categories for six of nine participants. From Figure 3, it is also clear that during the word generation task, features from the left MCA were chosen more frequently than features from the right MCA. Again, this meshes well with the observation that this task was lateralized to the left MCA.

As expected, lateralization was less prominent for the mental rotation task. Figure 3 shows that bilateral features were rarely selected for this task, while features from the left and right MCAs were chosen fairly equally, reflecting bilateral activation. When the mean of either MCA was selected, it was typically from 15–30 or 30–45 seconds. This could, perhaps, represent CBFV settling near the end of the state at a low value during rest or a high value during activation. When the slope of either MCA was selected, it was typically from 0–45 or 0–15 seconds. This may represent the general decreasing/increasing trend in CBFV during rest/activation, which is particularly pronounced at the beginning of each state. The frequent selection of the interval from 0 to 15 seconds suggests that slope features may be useful for shortening the BCI response time. Potential justification for this reasoning can be drawn from Figure 1, which shows a representative example of the CBFV trends in both MCAs during the rest state and both types of activation states. It is clear in this case that significant increases in CBFV occurred very early within both activation states.

### Influence of Respiratory Modulation

Prior to the experiment, no instructions were given to participants regarding the modulation of respiration. However, it was observed that some participants, either intentionally or unintentionally, modulated their respiration between rest and activation states. These modulations were apparent when classification was performed on respiratory data alone, as shown in Tables 2 and 3. These findings are of interest because respiratory modulations are known to affect CBFV [40]. However, classification using TCD data obtained significantly greater accuracies than classification using respiratory data for eight of nine participants for both tasks. This indicates that the results we have obtained are not merely the result of changes in respiration. Furthermore, it was shown by Szirmai et al [30] and by Hartje et al [35] that respiration-induced changes in CBFV tend to be bilateral. This suggests that we can reduce the impact of changes in respiration by using only bilateral features. The disadvantage of this approach is that we are likely to significantly diminish the attained accuracy for tasks which are weakly lateralized or unlateralized. When we restricted feature selection to bilateral features, this hypothesis was verified; we did incur a significant drop in accuracy for the mental rotation task. However, we maintained a very high accuracy for the word generation task. This provides further indication that the effects of respiratory modulation were not a significant factor during classification of the word generation task.

### Limitations

During this study, we used a duration of 45 seconds for both rest and activation states. This is comparatively long for BCI applications, and in practical usage would limit the data transmission rate. Such a lengthy duration was chosen due to the lack of pre-existing research into a TCD-based BCI, and the necessity of obtaining high classification accuracy. However, results from this study indicate that shorter durations would still allow for reliable detection of cognitive activity. In Figure 1, it is clear that significant increases in CBFV within the left and right MCAs occur soon after the onset of cognitive activity. Future studies should investigate the impact of shorter durations on classification accuracy.

In this study, the potential benefits of practice were not examined, as the participant only completed two sessions. Despite this, high classification accuracies were obtained. It is possible that participants could become even more accurate as they gained further proficiency with the required mental tasks. However, it is also possible that further practice could lead to habituation and a reduction in the cognitive activation caused by each mental task. Longer-term studies are needed to investigate these issues.

One difficulty associated with TCD is the presence of CBFV artifacts associated with movement. Significant head movement could cause the TCD probes to shift, resulting in momentary or persistent deterioration of the recorded signals. Body movement can also induce CBFV changes that may incorrectly be classified as activation, causing a false positive. These movements could also affect lateralization if they occurred during the performance of either mental task. However, such movements were observed in many participants during this experiment, and they did not prevent high classification accuracies. Consequently, we hypothesize that a TCD-based BCI would be robust to movement artefacts. Future studies should investigate this issue, as such movement artefacts are likely to be common during practical BCI usage.
